# MiR-26a-5p regulates cardiac fibroblasts collagen expression by targeting ULK1

**DOI:** 10.1038/s41598-018-20561-4

**Published:** 2018-02-01

**Authors:** Liling Zheng, Sihuang Lin, Chengyu Lv

**Affiliations:** Department of Cardiovascular Surgery, First Hospital of Quanzhou Affiliated to Fujian Medical University, Quanzhou, Fujian, China

## Abstract

MiRNA is a class of small non-coding RNA which has an important effect on posttranscriptional gene regulation. It can regulate the expression of the target gene at the mRNA level and further influence the protein level of the target gene. We found that ULK1 may be the target gene of miR-26a-5p, and ULK1 (unc-51 like autophagy activating kinase 1) is a key component in autophagy pathway. In this study, we overexpressed miR-26a-5p by transfecting miR-26a-5p mimic into cells and simultaneously inhibited miR-26a-5p by transfecting miR-26a-5p inhibitor into cells. We demonstrated that overexpression of miR-26a-5p can reduce the expression of ULK1 and collagen I, and decrease the activation of LC3-I to LC3-II. In contrast, inhibition of miR-26a-5p can increase the expression of ULK1 and collagen I, and increase the activation of LC3-I to LC3-II. The Dual-luciferase reporter assay showed that miR-26a-5p directly acted on the 3′UTR of ULK1 and thus affected the expression of ULK1. As such, our study demonstrated that miR-26a-5p might regulate the autophagy in cardiac fibroblasts by targeting ULK1, which may have an effect on cardiac fibrosis. To our knowledge, this is the first study that shows miR-26a-5p regulates the autophagic pathway in cardiac fibroblasts.

## Introduction

MicroRNAs (miRNAs) are a class of endogenous small non-coding RNAs about 22 bases in length. In 1993, Lee *et al*. found that small RNAs affect growth and development in *C elegans*^1^. Subsequently, more miRNAs were found. The number of confirmed miRNAs has reached 28645 from the mirBase database data as of June 2014, which are widely involved in regulation of organ development, cell proliferation, differentiation and apoptosis^2^. *In vivo*, miRNAs genes are first transcribed into the primary miRNA (pri-miRNA) in the nucleus, and then processed into stem-loop structure precursor miRNA (pre-miRNA) including about 70 nucleotides; in the cytoplasm, the pre-miRNA is cleaved to mature miRNA with about 22 nucleotides length^3^. As a Key factor in posttranscriptional regulation, miRNAs mainly affect the stability of transcripts^4^ leading to the changes of protein levels^5^. Studies have shown that miR-18/19 are involved in the regulation of extracellular matrix protein including connective tissue growth factor (CTGF) and thrombospondin-1 (TSP-1) and type I and type III collagen, and thus affect the process of cardiac fibrosis^6^. To date, accumulating studies have indicated that miRNAs are involved in the development of cardiovascular diseases and may become new therapeutic targets in prevention and treatment of cardiovascular disease^7^.

**Figure 1:**
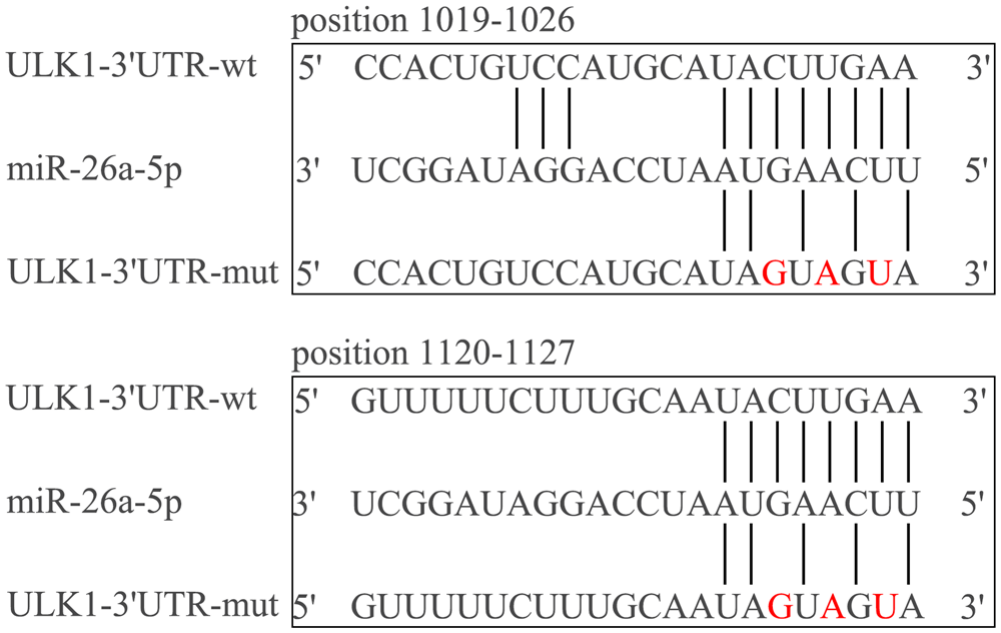
MiR-26a-5p binding sites and mutation sites. The specific binding sites of miR-26a-5p in ULK1 3′-UTR, mutation sites were designed according to this sequence.

Through MiRanda, TargetScan and other bioinformatics software, we found that MiR-26a-5p may target ULK1 (unc-51 like autophagy activating kinase 1), a key component of autophagy pathway. The possible sites of interaction have been shown in Fig. 1. Studies have shown that miR-26 can regulate cell autophagy in hepatocellular carcinoma^8^. ULK1 is a serine/threonine protein kinase, which could induce autophagy by enhancing the activity of the VPS34 complex and phosphorylating Beclin1^9^. The microtubule-associated protein 1 light chain 3 (LC3) participates in the formation of autophagosome membranes during autophagy, which have two forms called LC3-I and LC3-II, respectively. During autophagosome formation, LC3-I is conjugated to phosphatidylethanolamine to form LC3-II, the active form. LC3-II is a marker of autophagosome formation as it located on the autophagic membrane^10^. To date, little is known about the function of miR-26a-5p and its regulation of ULK1 in cardiac fibrosis. The purpose of present study is to investigate the function of miR-26a-5p in cardiac fibroblasts. Here we show that miR-26a-5p regulates the expression of ULK1 and subsequently affects autophagy in cardiac fibroblasts.

## Results

### ULK1 3′UTR mutation site design

The 3′-UTR sequence of ULK1 (NM_001108341.1) was found in the NCBI database, which contains the specific binding sites of miR-26a-5p. The mutant ULK1–3′UTR were designed according to this sequence. MiR-26a-5p and ULK1–3′UTR binding site and mutation sites as Fig. 1 showed.

### MiR-26a-5p acts directly on the ULK1 3′UTR region

**Figure 2:**
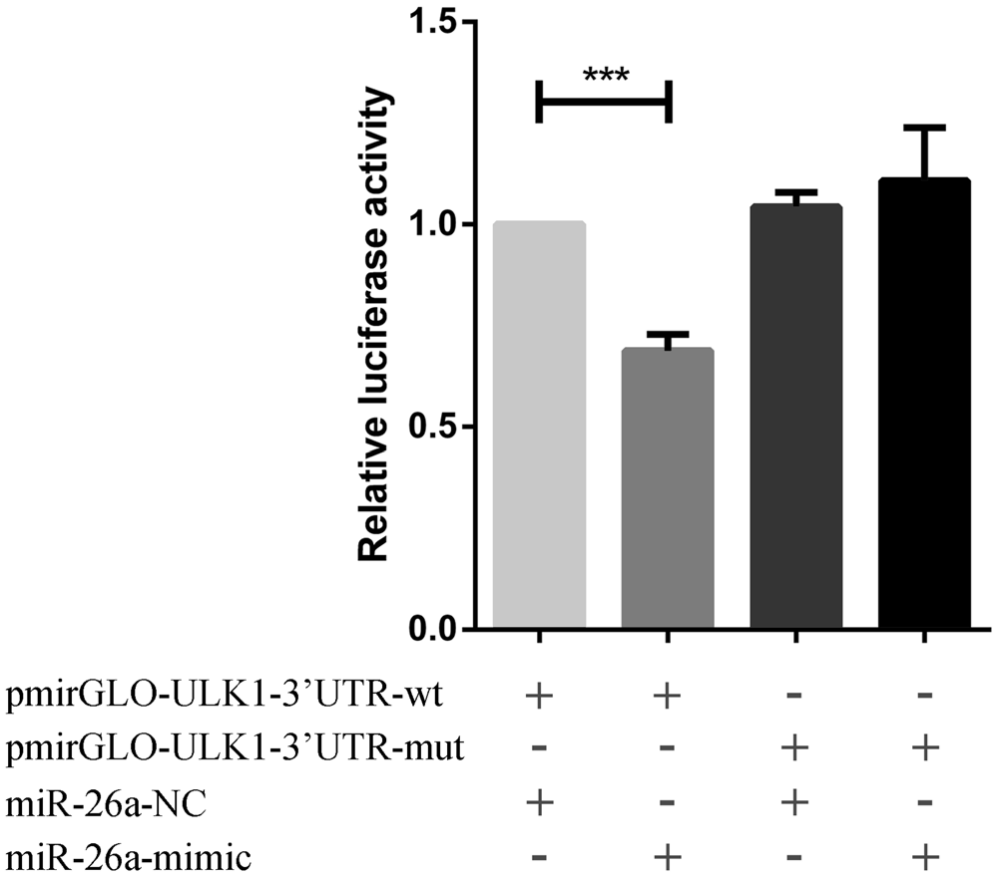
Dual-luciferase reporter assay. The reported plasmid in which the luciferase coding sequence is fused to ULK1–3′-UTR-Wild or ULK1–3′-UTR-Mutant and miR-26a-5p mimic. miR-26a-5p inhibitors were co-transfected into cardiac fibroblasts. Renilla luciferase activity was used as internal control to normalize Firefly luciferase activity. (*P < *0*.*05*) compared with NC group.

In order to verify whether the mRNA target of miR-26a-5p is ULK1, we cloned the rat ULK1–3′UTR fragment into dual luciferase reporter vector–pmirGLO and the recombinant plasmid was named pmirGLO-ULK1–3′UTR-wt. We also mutated the miR-26a-5p targeting sequence in above ULK1–3′UTR reporter plasmid, named as pmirGLO-ULK1–3′UTR-mut. With these two reporter plasmids, we performed luciferase assay in HEK293T cells. The ULK1–3′UTR reporter activity was significantly down-regulated when co-transfected with miR-26a-5p mimic compared with non-targeting control miRNA (*P* < *0*.*05*) . Moreover, miR-26a-5p mimic had no effect on the reporter activity of pmirGLO-ULK1–3′UTR-mut (Fig. 2). Collectively. These data indicated that ULK1 mRNA is the target of miR-26a-5p.

### Overexpression or inhibition of miR-26a-5p expression regulates ULK1 mRNA expression

**Figure 3:**
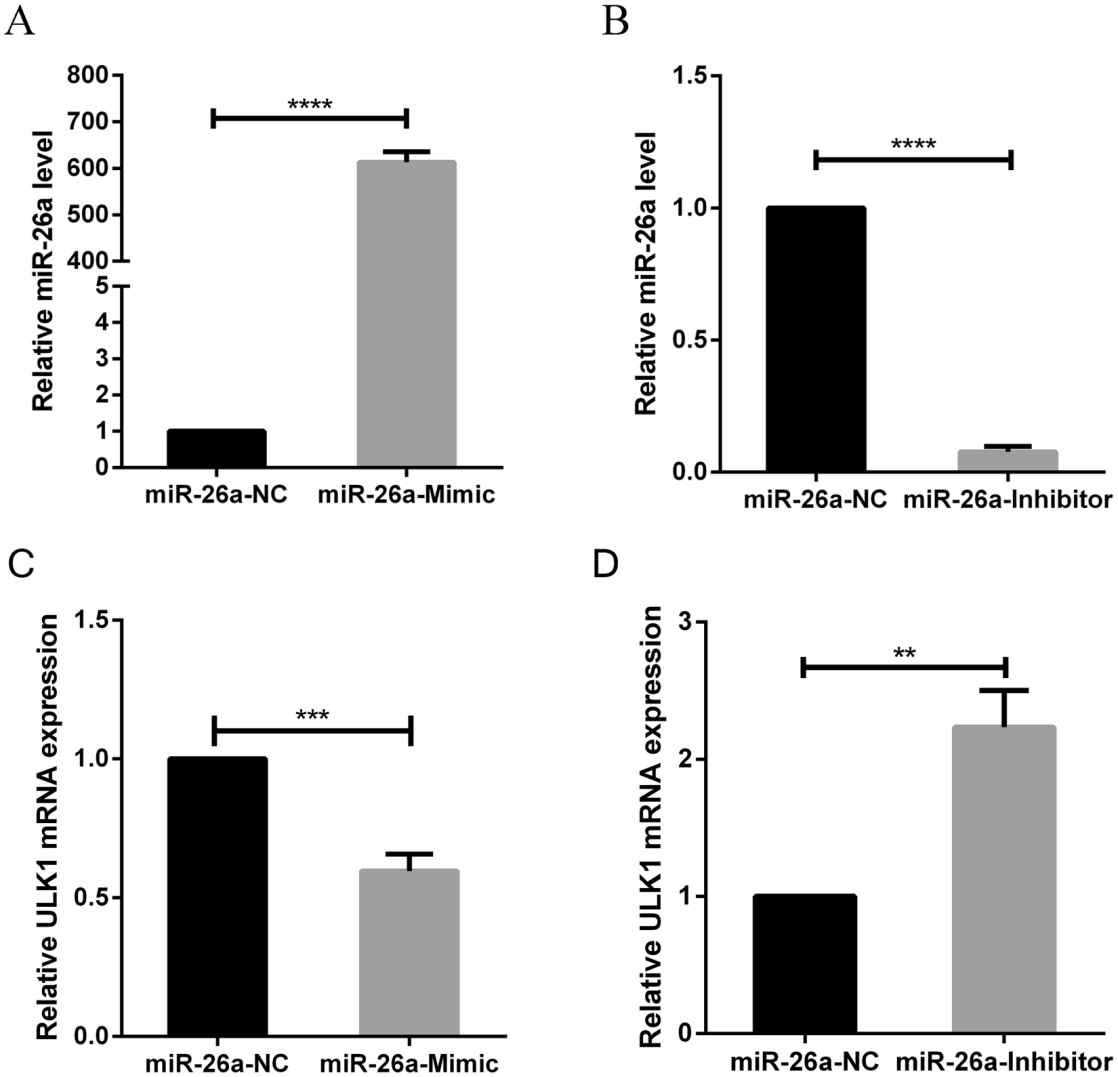
Effects of miR-26a-5p on the expression level of ULK1 mRNA. Quantitative real-time PCR in detection miR-26a-5p level after (**A**) transfection of miR-26a-5p mimic or (**B**) inhibitor into cardiac fibroblasts. (*P < *0*.*05*) compared with NC group. ULK1 level after transfection of miR-26a-5p mimic (**C**) or inhibitor (**D**) into cardiac fibroblasts. (*P < *0*.*05*) compared with NC group.

To confirm that miR-26a-5p regulates endogenous ULK1 mRNA, we transfected cardiac fibroblasts with miR-26a-5p mimic and its inhibitor, and detected miR-26a-5p and ULK1 mRNA expression levels by quantitative real-time PCR. The expression level of miR-26a-5p was significantly up-regulated when cardiac fibroblasts were transfected with miR-26a-5p mimic (*P* < *0*.*05*) compared with Control (Fig. 3A). Conversely, the expression level of miR-26a-5p was significantly down-regulated when cardiac fibroblasts were transfected with miR-26a-5p inhibitor (*P* < *0*.*05*) compared with Control(Fig. 3B). The expression of ULK1 mRNA was significantly down-regulated when cardiac fibroblasts were transfected with miR-26a-5p mimic (*P* < *0*.*05*) (Fig. 3C). Conversely, the expression of ULK1 mRNA was significantly up-regulated when cardiac fibroblasts were transfected with miR-26a-5p inhibitor (*P* < *0*.*05*) (Fig. 3D). These data suggest that miR-26a-5p can regulate the expression of endogenous ULK1 mRNA in cardiac fibroblasts.

### Overexpression or inhibition of miR-26a-5p expression can regulate the expression of ULK1 protein and affect autophagy in cardiac fibrosis

**Figure 4:**
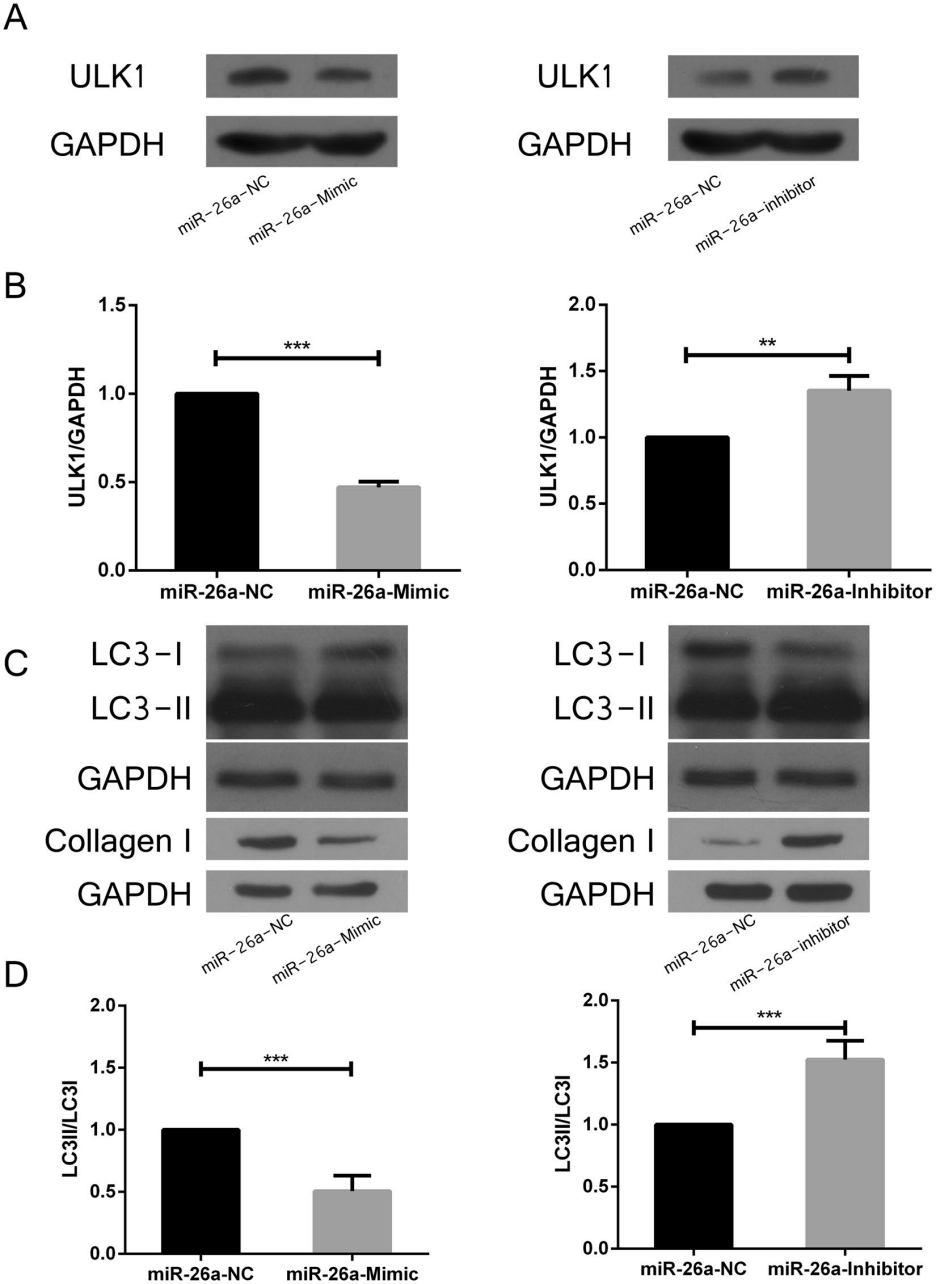
Effects of miR-26a-5p on the expression level of ULK1, Collagen I and LC3 protein. Western blot analysis of the expression level of ULK1 protein after transfection of miR-26a-5p mimic (**A**) or inhibitors (**B**) into cardiac fibroblasts. The expression level of collagen I and the activation of LC3-I to LC3-II level after transfection of miR-26a-5p mimic or inhibitors (**C**,**D**) into cardiac fibroblasts (*P < *0*.*05*) compared with NC group.

To determine the effect of miR-26a-5p on the expression level of ULK1, Collagen I and LC3 protein, we transferred miR-26a-5p mimic and inhibitor in cardiac fibroblasts, and detected the expression levels of ULK1, Collagen I and LC3 protein by western blot. The ULK1 protein level was significantly down-regulated by miR-26a-5p mimic compared with Control. In contrast, ULK1 protein level was significantly up-regulated by miR-26a-5p inhibitor (Fig. 4A,B). With the increased expression of miR-26a-5p, Collagen I and the conversion of LC3-I to LC3-II was decreased, whereas the decreased expression of miR-26a-5p increased expression of Collagen I and conversion of LC3-I to LC3-II (Fig. 4C,D). These data indicated that miR-26a-5p could regulate the expression of ULK1 protein and affect cell autophagy and cardiac fibrosis.

### Overexpression or inhibition of miR-26a-5p expression do not change the expression of Collagen I and LC3 mRNA

**Figure 5:**
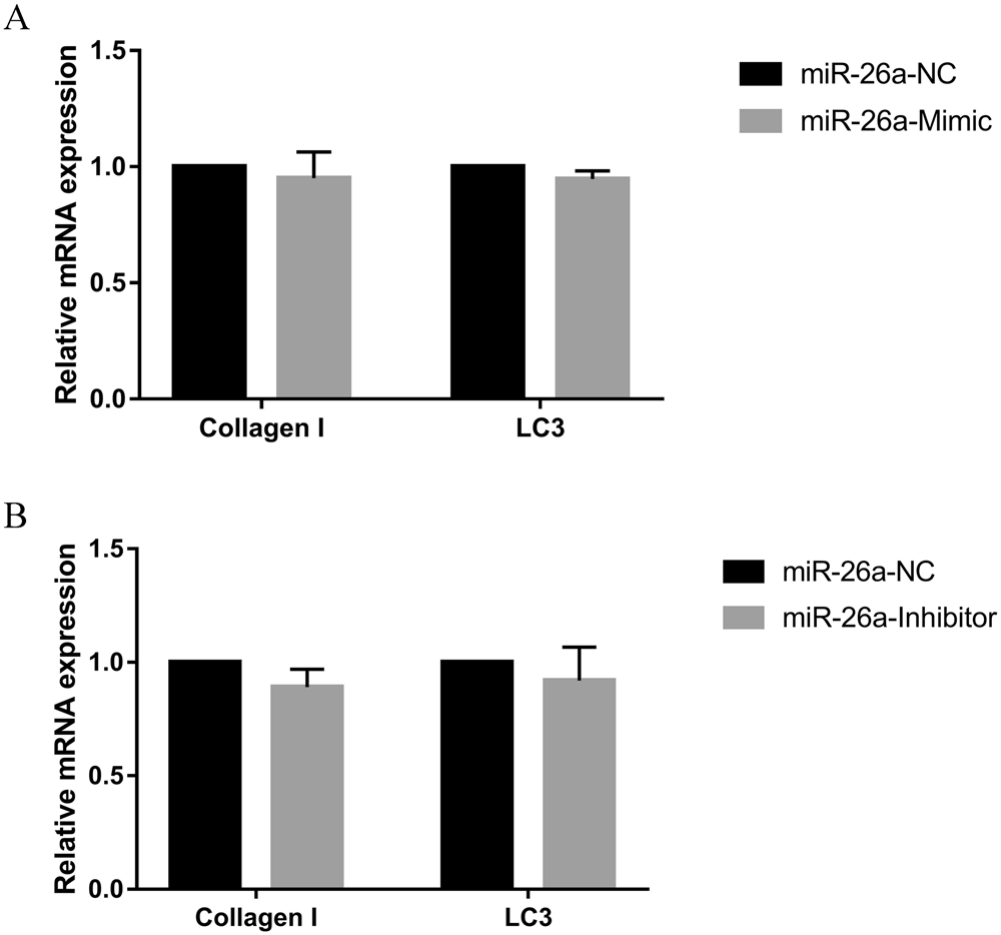
Effects of miR-26a-5p on the expression level of Collagen I and LC3 mRNA. Quantitative real-time PCR in detection the expression level of Collagen I and LC3 mRNA after transfection of miR-26a-5p mimic or inhibitor into cardiac fibroblasts. The expression of Collagen I and LC3 mRNA were no obvious changed when cardiac fibroblasts were transfected with miR-26a-5p mimic or inhibitor (*P* > *0*.*05*).

To determine whether miR-26a-5p regulates Collagen I and LC3 mRNA expression, we transfected cardiac fibroblasts with miR-26a-5p mimic and its inhibitor, and detected Collagen I and LC3 mRNA expression levels by quantitative real-time PCR. The expression of Collagen I and LC3 mRNA were no obvious changed when cardiac fibroblasts were transfected with miR-26a-5p mimic or inhibitor (*P* < *0*.*05*) (Fig. 5). These data suggest that miR-26a-5p does not directly change the expression of Collagen I and LC3 mRNA.

## Discussion

Cardiovascular disease (CVD) is the number 1 cause of death in China and worldwide. Heart failure is the final stage in all kinds of cardiovascular diseases. The main pathological changes of heart failure are ventricular remodeling and fibrosis, which are accompanied in the whole process of heart failure. The main cellular components of the heart are cardiac fibroblasts, myocytes, endothelial cells, and vascular smooth muscle cells. Research data have shown that fibroblasts accounted for 27% and 64% in mice and rat hearts^11^. Cardiac fibroblasts are essential to maintaining cardiac function but they are also the main determinant of cardiac fibrosis. Excessive deposition of extracellular matrix leads to cardiac fibrosis and the main source of extracellular matrix is cardiac fibroblasts^12^. Therefore, in our study, we chose to study primary cardiac fibroblasts as a good cellular model of cardiac fibrosis. Numerous studies have shown that varieties of miRNAs are involved in the regulation of cardiac fibrosis. Roy and his team have found that the expression of miR-21 in cardiac fibroblasts was elevated during myocardial ischemia-reperfusion in mice, and that miR-21 is associated with the inhibition of cardiac fibroblast apoptosis and the regulation of infarct repair^13^. The microarray analysis of miRNAs in the TAC model showed that the expression of multiple miRNAs is changed within two weeks after surgery, which included the down-regulation of miR-26a-5p expression on 14 days postoperatively. The results suggest a strong connection with the expression of miRNAs and the regulation of cardiac hypertrophy^14^. There is a study showed that miR-29 (includes miR-29a, miR-29b, miR-29c) plays a negative role in cardiac fibrosis, which regulates genes including Collagen I, Collagen III, Elastin, and Fibronectin. Animal experiments and cell experiments showed that the expression of Collagen, Elastin, and Fibronectin was up-regulated when miR-29 was down-regulated, whereas the expression of collagen, elastin, and fibronectin was down-regulated when miR-29 was up-regulated^15^. Our study showed that when the expression of miR-26a-5p changed, the protein expression of Collagen I also changed, which suggested that miR-26a-5p could affect cardiac fibrosis.

Autophagy is a process of cell self-digestion, the function of which is to degrade unwanted or dysfunctional components in cells, re-use it, and keep cells in a state of dynamic equilibrium^16^. Autophagy plays a critical role in heart disease, by either protecting heart from disease or promoting the development of heart disease. For examples, overexpression of mTOR in mice inhibited autophagy and improved cardiac function, which suggest that autophagy may not play a protective role in the heart function^17^. Similarly, excessive autophagy promotes the development of heart failure as observed in beclin1 overexpression mice^18^. However, other researches have indicated that autophagy plays a protective role in heart failure by removing damaged proteins^19^. Simonson found that DDiT4L promotes autophagy and inhibits pathological cardiac hypertrophy in response to stress by inhibiting mTOR-signaling pathways^20^.

We used bioinformatics software to predict that the target gene for miR-26a-5p may be ULK1. The ULK1 gene is one of the key factors for autophagy initiation and plays a cardinal role in the regulation of autophagy^21^. The ULK1 gene is regulated by a variety of factors. For instance, AMPK could phosphorylate and activate ULK1, and thus modulate the cell autophagy^22^. In our study, we showed that the expression levels of ULK1 mRNA and protein were significantly changed when the expression of miR-26a-5p changed, indicated that miR-26a-5p could regulate ULK1. In the process of autophagy, the level of LC3-II protein is positively correlated with autophagic activity, and therefore can be regarded as a marker of autophagy^23^. We confirmed that the conversion of LC3-I to LC3-II was significantly changed when up-regulated or down-regulated the expression of miR-26a-5p, instead of directly changing the expression of LC3 mRNA, suggested that miR-26a-5p can regulate autophagic activity likely through the interaction of miR-26a-5p with ULK1 mRNA.

In summary, Our studies provided a valuable clue for developing a prospective therapeutic target for control of heart fibrosis in the future, although the detail mechanisms of miR-26a-5p, ULK1 and cardiac fibrosis remain to be fully elucidated.

## Materials and Methods

### The acquisition and culture of primary cardiac fibroblasts

All animal care and experimental procedures were approved by the Experimental Animal Research Committee of Fujian Medical University, and in strict accordance with the recommendations in the Guide for the Care and Use of Laboratory Animals of the NIH. Hearts were removed from neonatal rats (1~3 days) which provided by Fujian Medical University Experimental Animal Center (Fuzhou, China), cut into 0.5~1.0 mm^3^ small pieces and digested with 0.25% trypsin (Gibco, USA) to get single cell suspension. The cells were filtered through 100 uM cell strainer to remove tissue debris and then collected by centrifugation (1000 rpm/min, 10 min). Cells were resuspened in DMEM (Hyclone, USA) medium containing 10% fetal bovine serum (PAN, Germany) and placed into cell culture dishes for 40 min. Then cell dishes were washed gently with PBS buffer for three times, and added with fresh DMEM medium to culture primary cardiac fibroblasts.

### Construction of ULK1 3′-UTR and mutants ULK1 3′-UTR

The miR-26a-5p sequence (MIMAT0000796) was found in the miRBase database, the 3′-UTR sequence of ULK1 (NM_001108341.1) was found in the NCBI database, which contains the specific binding sites of miR-26a-5p. The mutant ULK1–3′UTR were designed according to this sequence. The primers of the ULK1 3′-UTR were as follows: forward, 5′-CTAGCTAGCTAGAGTAGGGACAGTCGTCGTG-3′; reverse, 5′-GCTCTAGAGCTTCAGTGAGCAGGTTTGG-3′. To construct a double luciferase reporter vector, the fragment of ULK1 3′-UTR was ligated into a dual luciferase reporter vector–pmirGLO. Using this as a template, mutation of the miR-26a-5p targeting sites was performed by site-directed mutagenesis. Mutation sites are shown in Fig. 1.

### Cell transfection

When the cell culture reached 30 ~ 50% confluence, medium was replaced with serum-free DMEM 1 hour before transfection and cells were transfected with X-tremeGENE siRNA reagents (Roche, Switzerland) according to manufacturer’s instructions. MiR-26a-5p mimic and inhibitor were purchased from GenePharma (Shanghai, China) .The sequence of miR-26a-5p mimic NC: 5′-UUCUCCGAACGUGUCACGUTT-3′, the sequence of miR-26a-5p inhibitor NC: 5′-CAGUACUUUUGUGUAGUACAA-3′.

### Dual-luciferase reporter assay

HEK-293T (Shanghai, China) cells were seeded in 12-well plates and the recombinant ULK1 3′-UTR (WT or mutant reporter plasmids) and miR-26a-5p (mimic or inhibitor) were transfected with X-tremeGENE siRNA transfection reagent (Roche, Switzerland). Luciferase activity was detected by the firefly luciferase reporter assay kit (Promega, USA) instructions at 48 h post transfection.

### RNA isolation and quantitative real-time PCR

The expression level of miR-26a-5p mimic, inhibitor and ULK1 mRNA were detected by quantitative real-time PCR. Primary cardiac fibroblasts were transfected with miR-26a-5p mimic, inhibitor and their controls, respectively. Total RNA was extracted using TRIzol reagent (ambion, USA) at 48 h post transfection. One μg total RNA was reversely transcribed into cDNA, and subjected to quantitative real-time PCR (Dalian, China). The miR-26a-5p specific primers were purchased from RiboBio Biotech (miRQ0000796–1–1, Guangzhou, China), and U6 as the Reference gene. The primers of the ULK1 were as following: forward, 5′-CCCCAACCTTTCGGACTT-3′, reverse, 5′-CCAACAGGGTCAGCAAACTC-3′, the primer of Collagen I: forward, 5′-ACGCATGAGCCGAAGCTAAC-3′, reverse, 5′-AGGGACCCTTAGGCCATTGT-3′ the primer of LC3: forward, 5′-CGCACCTTCGAACAAAGAG-3′, reverse, 5′-CTCACCCTTGTATCGTTCTATTATCA-3′, the primer of endogenous control GAPDH: forward, 5′-CGCATTGCCAGACATATCAGC-3′, reverse, 5′-AGGTGAAGCAGGCTCAATCAA-3′. The relative expression levels was calculated according to equation 2^^− ΔΔCT^.

### Protein extraction and Western blot

The expression of ULK1 and LC3 protein was detected by Western blot. Total protein was extracted from cardiac fibroblasts by the Western & IP cell lysis buffer (Beyotime, China) and protein concentration was determined by BCA method. For Western blotting, equal amounts of protein samples (50 μg) were separated by sodium dodecy sulfate-polyacrylamide gel electrophoresis (SDS-PAGE) and transferred onto a polyvinylidene fluoride (PVDF) membrane (AmershamTMHybondTM, Germany). The PVDF membrane was blocked with 5% bovine serum album (amresco, USA) for 1 h and then incubated with primary antibody at 4 °C overnight. Primary antibodies were: rabbit anti-ULK1 (Abcam, ab128859) diluted 1:1000; rabbit anti-LC3 (Cell Signaling, #4108) diluted 1:1000; rabbit anti-Collagen I (Abcam, ab34710) diluted 1:1000; rabbit anti-GAPDH (Cell Signaling, #5174) diluted 1:1000. On the next day, the membranes were washed with TBS-T (0.1% Tween-20) and further incubated with HRP-linked secondary antibody (Cell Signaling, #7074) diluted 1:2000. The membranes were then visualized by chemiluminescence BeyoECL Plus (Beyotime, China).

### Statistical analysis

The experimental data were expressed as mean ± SEM, and analyzed with SPSS 18.0 statistical software. Comparisons were made using 2-tailed student’s t test between two group and with ANOVA among 3 groups or more. Any P value less than 0.05 was considered as statistically significant.

## Electronic supplementary material

supplementary figures
