# Supplementary material for: MiR-26a-5p regulates cardiac fibroblasts collagen expression by targeting ULK1
Source: Sci Rep. 2018 Feb 1;8:2104. doi: 10.1038/s41598-018-20561-4 (PMC5794903; doi:10.1038/s41598-018-20561-4)
Supplement: Supplementary file 1 — supplementary figures [file 41598_2018_20561_MOESM1_ESM.docx]

**MiR-26a-5p regulates cardiac fibroblasts collagen expression by targeting ULK1**

Liling Zheng*, Sihuang Lin, Chengyu Lv

Supplementary figures：

Cardiac fibroblast morphology：This is the morphology of the cells used in our study


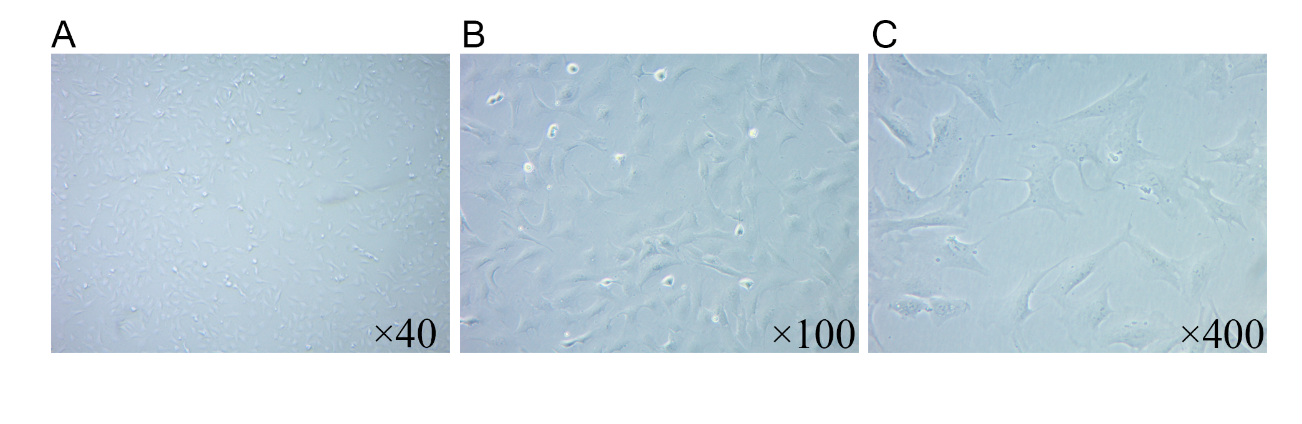


Effects of miR-26a-5p on the expression level of ULK1 protein：This is the original image of Figure 4


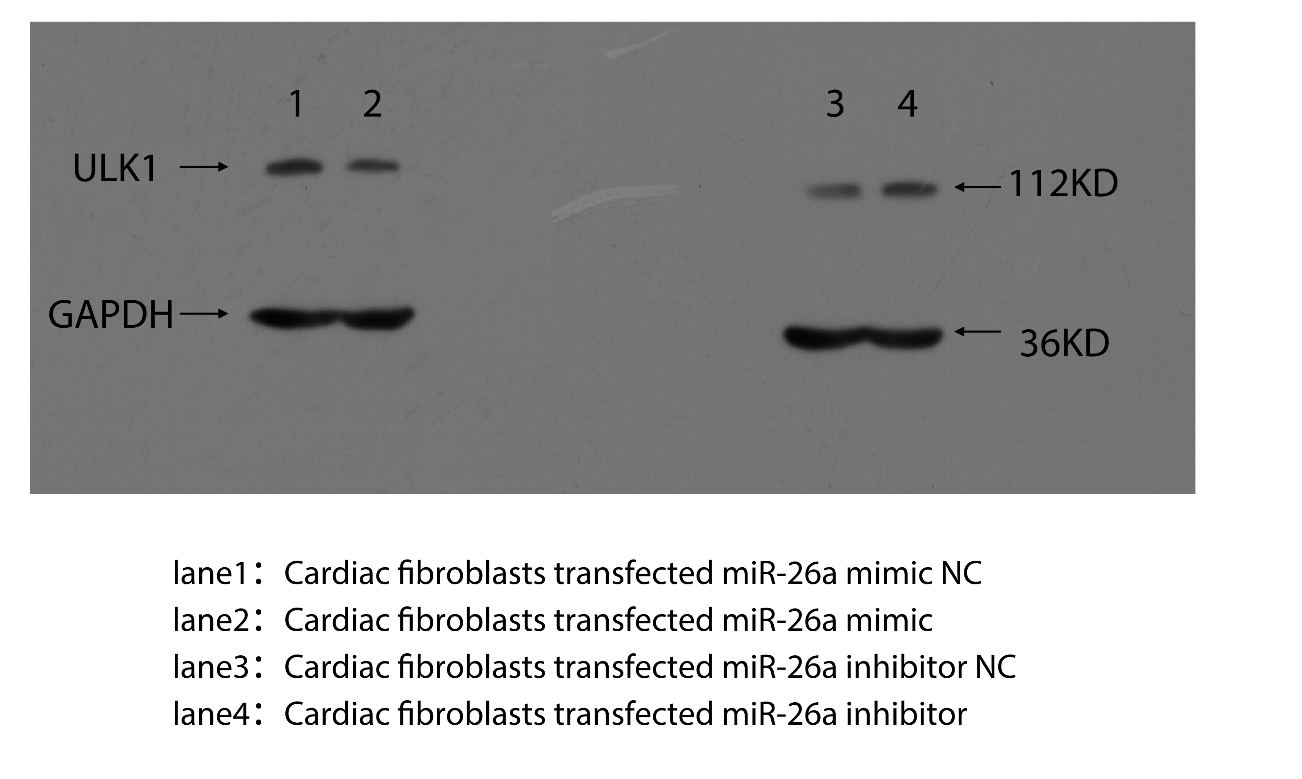


Effects of miR-26a-5p on the expression level of LC3 protein：This is the original image of Figure 4





Effects of miR-26a-5p on the expression level of Collagen I protein：This is the original image of Figure 4
